# Induction of Antigen-Specific Tolerance in Autoimmune Diabetes with Nanoparticles Containing Hybrid Insulin Peptides

**DOI:** 10.3390/biomedicines9030240

**Published:** 2021-02-27

**Authors:** James E. DiLisio, Kathryn Haskins

**Affiliations:** Department of Immunology and Microbiology, University of Colorado School of Medicine, Aurora, CO 80045, USA; james.dilisio@cuanschutz.edu

**Keywords:** autoimmunity, NOD mouse, T cells, hybrid insulin peptides (HIPs), induction of tolerance, nanoparticles

## Abstract

Autoreactive T cells are thought to orchestrate the onset and progression of autoimmune diabetes. Key cognate antigens of these diabetogenic T cells include hybrid insulin peptides, formed by the fusion of insulin fragments to cleavage products of other β-cell granule proteins. Here we review initial work exploring tolerance induction to a hybrid insulin peptide using a biodegradable, nanoparticle delivery system in non-obese diabetic (NOD) mice. The immune phenotype(s) and possible mechanism(s) behind antigen-specific tolerance induction were dissected with a disease transfer model using transgenic autoreactive mouse T cells. Treatment of NOD mice with peptide-coupled nanoparticles appeared to have a dual function in preventing diabetes onset, inducing anergy in effector T cells and enhancing the activity of regulatory T cells. Importantly, the ratio of these two cell types in the pancreas was pushed toward tolerance. Antigen-specific tolerance induction to hybrid insulin peptides has the translational potential to preserve islet β-cells in new-onset or at-risk patients and prevent recurrent autoimmunity in transplant patients.

## 1. Introduction

Type 1 diabetes (T1D) is an autoimmune disease in which the insulin-producing β-cells in the pancreatic islets are destroyed, resulting in hyperglycemia. Autoreactive T cells directed toward islet antigens play an important role in this process, entering the pancreas at an early stage and contributing substantially to the infiltration of immune cells in the islets or insulitis [1,2,3,4]. The only available ongoing treatment on a cost-effective and reasonable basis for most patients is insulin replacement therapy; however, complications such as kidney and vascular disease still occur in treated patients. Even with important advances in islet transplantation and strategies for inducing tolerance to grafts, recurrent immune activation to β-cells is a major hurdle in long-term islet graft survival. Currently, a broad immunosuppressive regimen is used to control graft rejection but leaves the patient vulnerable to a myriad of common pathogens. Novel therapeutic approaches that can arrest disease onset or aid islet transplantation without the need for broad immunosuppression are under intense investigation. In this review, we discuss recent advances in the use of nanoparticle antigen transport as a viable approach for effective long-term treatment of T1D.

## 2. Autoreactive T Cells and Their Cognate Peptide Antigens

Identifying key T cell clones that drive disease or act as biomarkers for disease initiation/progression has been a major goal in T1D research. The spontaneously autoimmune non-obese diabetic (NOD) mouse model has been the most widely used model system for this work. In our early studies on the role of CD4 T cells in the NOD mouse, we isolated and characterized a panel of islet-reactive, diabetes-inducing CD4+ T cell clones, the BDC (Barbara Davis Center) panel [5,6,7]. The prototype T cell clone from this panel, BDC-2.5, responds robustly to islet cells in vitro by the production of IFNγ and is highly diabetogenic when transferred to young mice [8]. Subsequently, the BDC-2.5 T cell receptor (TCR) was sequenced and used to make the BDC-2.5 TCR transgenic (TCR-Tg) mouse model [9], which has been widely used by investigators to study the role of autoreactive T cells in the pathogenesis and regulation of autoimmune diabetes [7]. One of the primary ways in which we have used the BDC panel of T cell clones is to define the peptide ligands for autoreactive T cells in diabetes.

The identification of islet antigens that activate autoreactive T cells has been a highly pursued research goal in T1D. The BDC T cell clones were originally selected for antigen reactivity on whole islet cells and were subsequently maintained and tested in antigen response assays on NOD islet cells. We first investigated various candidate antigens, including insulin, insulinoma antigen-2 (IA-2), and glutamic acid decarboxylase (GAD), that had been previously identified as autoantibody targets in diabetes but found that extracts and/or peptides of these proteins did not activate BDC T cells [7]. A variety of approaches and efforts over many years were applied to identify T cell antigens and ultimately, a combination of biochemical and mass spectrometric techniques led to the identification of chromogranin A (ChgA) and islet amyloid polypeptide (IAPP) as potential sources of the peptide ligands for several of the BDC T cell clones [10]. Subsequent work showed that the actual antigens were peptide hybrids consisting of an insulin C-peptide fragment covalently linked either to a natural cleavage product of ChgA (WE14) or to a cleavage product of pro-IAPP and that most of the BDC T cell clones reacted to one or the other of these products [11]. The hybrid insulin peptide (HIP) consisting of a C-peptide sequence linked to WE14 was found to be the ligand for the BDC-2.5 clone and was thus named the 2.5HIP. It was hypothesized that these neo-antigenic HIPs are generated in islet β-cells through a novel post-translational modification process, likely a transpeptidation reaction. However, since mass spectrometry has shown that HIPs are present in normal islets of mice and humans [12], the question of how these peptides are processed and presented to T cells remains an important issue. NOD mice have a relatively high prevalence of HIP-reactive T cells in blood and spleen, and in the pancreas, their numbers increase over the course of disease progression [13]. As anticipated, these T cells exhibit a Th1 inflammatory phenotype, secreting IFNγ and TNFα [13]. T cells reactive to HIPs have also been detected in the islets of deceased human T1D donors and in the peripheral blood of new-onset T1D patients, suggesting their involvement in disease initiation [14].

## 3. PLG Nanoparticles for Induction of Antigen-Specific Tolerance

There have been several reports of tolerance induction in the NOD mouse model through the delivery of autoantigens as tolerogens, including whole insulin and a Glutamic Acid Decarboxylase 65 (GAD65) peptide [15]. Unfortunately, when these immunotherapies were transitioned to human clinical trials, there was little efficacy in generating tolerance against insulin [16] or GAD65 [17], and no long-term preservation of islet function. A more recent approach involving the use of biodegradable nanoparticles has shown promise in both animal models and in human trials. The FDA-approved, biodegradable poly(lactide-co-glycolide) nanoparticles (PLG-NPs) have been shown to safely transport peptides for antigen-specific tolerance induction and disease remission in Th1/Th17-mediated animal models of experimental autoimmune encephalomyelitis (EAE) [18] and T1D [19]. PLG-NPs have also shown efficacy in a Th2-mediated animal model of allergic airway inflammation both pre- and post-sensitization, implying a broad mechanism for tolerance induction [20].

Studies in the murine model of EAE [18] showed that upon intravenous (i.v.) delivery, PLG-NPs were phagocytized by marginal zone macrophages (MZMs), which express the class A scavenger receptor, MARCO (macrophage receptor with collagenous structure), in the spleen and liver. Uptake and subsequent induction of tolerance was shown to be dependent on MARCO [18], a conclusion supported by experiments in which MARCO-deficient mice were unable to generate tolerance in response to antigen-coupled PLG-NPs. In vitro experiments demonstrated that antigen-presenting cells treated with antigen-loaded PLG-NPs showed a decreased expression of co-stimulatory molecules (CD80, CD86) and the inflammatory cytokine, IL-12, coupled with an increase in PD-L1 expression. Antigen-presenting cells with this phenotype, i.e., capable of presenting antigens in the absence of co-stimulation and presence of PD-L1, are known to drive T cell anergy [21,22]. Delivery i.v. of tolerogenic antigens to MZMs may be essential to facilitate systemic and organ-specific tolerance to autoantigens in the islets of the pancreas.

## 4. Induction of Tolerance with 2.5HIP-Nanoparticles in A Disease-Transfer System

To assess the efficacy and mechanisms of tolerance induced by 2.5HIP-conjugated nanoparticles (2.5HIP NPs), an adoptive transfer model was used in which BDC-2.5 Tg T cells (from the BDC-2.5 TCR-Tg mouse) were administered i.v. to NOD.*scid* mice. As NOD mice on the *scid* background (NOD.*scid*) lack an intact immune system and do not develop disease, i.v. introduction of autoreactive BDC-2.5 Tg T cells (pre-activated in vitro with peptide antigen and IL-2) provides a robust model of disease transfer. Following transfer, 100% of recipient mice develop overt diabetes within an average of 10 days. This model allows for the analysis of tolerance induction by NPs using a largely clonal population of diabetogenic CD4 T cells within a short time frame. In a previous study, the Miller group used BDC-2.5 Tg T cells to transfer disease and as a tolerogen, p31, a peptide mimotope that activates BDC-2.5 T cells and can substitute for the actual T cell ligand [19]. The 2.5HIP had not yet been identified as the ligand for BDC-2.5 at the time of this study. It was found that i.v. administration of p31-associated PLG nanoparticles could induce tolerance and prevent diabetes onset in recipient NOD.*scid* mice for up to two months [19]. The authors found that there were decreased numbers of effector T cells (Teff) expressing IFNγ, IL-2, and GM-CSF in the pancreas of p31 PLG-NP-treated animals. There were also increased numbers of FOXP3+ regulatory T cells (Tregs) in both the pancreas and spleen of treated animals compared to controls. Additionally, levels of IL-10, a regulatory T cell cytokine, were dramatically increased in treated animals [19]. Antigen-specific tolerance was at least in part dependent on negative signaling through CTLA4 and PD-1. The authors concluded from this study that Tregs are important not only for tolerance induction in this model but also for its long-term maintenance.

In collaboration with the Miller group, we carried out tolerance induction experiments in the BDC-2.5 adoptive transfer model using the 2.5HIP neo-antigen coupled to PLG-NPs (2.5HIP NPs) [23]. Treatment with 2.5HIP NPs prevented disease onset for at least two months in 80% of animals. To characterize the mechanistic aspects of this tolerance induction, we investigated phenotypic changes after treatment with 2.5HIP NPs in both effector T cells and Tregs. The 2.5HIP tetramer-positive (tet+) T cells were isolated from the pancreas of NP-treated animals and were stimulated with the 2.5HIP ex vivo prior to intracellular staining for inflammatory cytokines and T subset–specific transcription factors. We found that INFγ and TNFα production by 2.5HIP tet+ cells in tolerized animals was dramatically reduced. Examination for expression of the Th1 master regulator T-bet after treatment revealed a reduction in IFNγ+ T-bet+ T cells whereas numbers of Foxp3+ T-bet+ T cells were increased, hinting at a potential reversal of effector to regulatory phenotype. The 2.5HIP tet+ Foxp3+ Tregs also exhibited some surface phenotypic changes including decreased levels of CD127, a marker that inversely correlates with Foxp3+ expression on regulatory T cells. Nearly all Tregs in the pancreas of treated animals expressed CD103 integrin at the eight-week time point, implying retention of these regulatory cells in islets. Induction of tolerance with 2.5HIP NPs greatly reduced the ratio of Teff/Tregs in both islets and spleen to nearly equal proportions, whereas Teffs outnumbered Tregs nearly 10:1 in control animals [23]. These data suggest that induction of tolerance with 2.5HIP NPs reduces, and potentially reverses, the effector phenotype of autoreactive Teff cells while activating antigen-specific Tregs.

**Figure 1:**
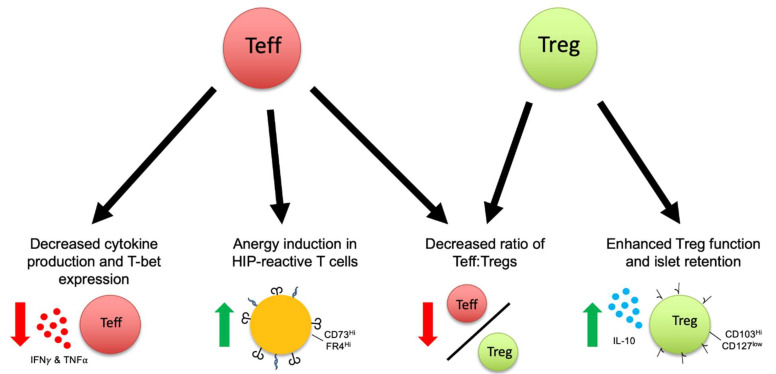
Phenotypic changes in both effector (Teff) and regulatory (Treg) T cells after antigen-specific tolerance induction with a hybrid insulin peptide conjugated to poly(lactide-co-glycolide) nanoparticles. Green arrows indicate an increase, Red arrows indicate a decrease; black arrows signify different effects on T cells after nanoparticle treatment. Line between Teff/Treg indicates the ratio of Teff to Treg.

To further clarify the mechanism by which Teff become tolerized, CD4+ Foxp3− cells were sorted after treatment for RNA sequencing. Transcriptional analysis revealed a highly anergic phenotype with overexpression of *Rnf128* (encoding GRAIL), *Egr2*, *Egr3*, and the *Egr3* ligand *Spry1* [23]. Genes involved in T cell exhaustion, such as those encoding for LAG3 and PD-1, were also markedly upregulated. The sequencing data highlight congruent transcriptional networks of both anergy and exhaustion in tolerized Teff cells. Surface staining two weeks after 2.5HIP NP treatment showed that the anergic phenotype was validated by an increased proportion of CD44^hi^ Foxp3− Teff cells co-expressing high levels of immunosuppressive proteins CD73 and FR4. The percentage of CD73^hi^, FR4^hi^ Teff cells further increased from 2 to 8 weeks, implying continued anergic signaling [23]. Whether the anergy/exhaustion induced in these cells results in eventual clonal deletion is unknown but reduced numbers of these cells over time (see above) suggest this possibility. Taken together, tolerance induction with the 2.5HIP NPs effectively targeted both sides of the disturbed Teff/Treg balance, as summarized in Figure 1, by driving anergy in Teffs and expanding Tregs and their functionality in an antigen-specific manner.

## 5. Summary and Future Directions

Delivery of HIPs coupled to PLG-NPs generates a robust tolerogenic T cell response and has been shown to at least delay disease onset in an adoptive transfer model of autoimmune diabetes. This therapy works in two ways, first by inducing anergy/exhaustion in autoreactive effector T cells and second, by expanding regulatory T cell populations, ultimately altering the ratio of Teff/Treg in islets to favor a state of tolerance. Ongoing work in our lab exploring tolerance induction by 2.5HIP NPs in a NOD islet transplant model also appears to be promising as initial data indicate treatment with 2.5HIP NPs can significantly delay graft rejection when compared to controls. This work further suggests that there may be a bystander tolerance effect wherein tolerization with one antigen may in turn lead to suppression of T cells reactive to other autoantigens.

There are also preliminary indications that PLG-NPs will be useful for the induction of antigen-specific tolerance in human subjects. The translational potential of antigen-specific PLG-NPs is indicated by promising results from a recent clinical trial in which gliadin-containing PLG-NPs were administered to patients with celiac disease. The trial showed that tolerization of patients with gliadin associated with PLG-NPs diminished both gliadin-specific CD4+ T cell IFNγ production and gut histological scores after a gluten challenge [24]. This represents one of the first attempts of antigen-associated nanoparticle-induced tolerance in a human disease and has promising implications for the translation of the PLG-NP delivery platform to autoimmune diseases like T1D. Looking to the future and precision medicine, due to the apparent heterogeneity of peptide reactivity in T1D patients as well as in NOD mice, the most effective therapy may require induction of tolerance to multiple antigens, perhaps two or more hybrid insulin peptides, for durable preservation of islet function. Antigen-specific tolerogenic therapies with HIPs may also provide greater potential for the long-term survival of transplanted islets.

## Data Availability

Not applicable.
